# Metabolic Insight into Glioma Heterogeneity: Mapping Whole Exome Sequencing to In Vivo Imaging with Stereotactic Localization and Deep Learning

**DOI:** 10.3390/metabo14060337

**Published:** 2024-06-16

**Authors:** Mahsa Servati, Courtney N. Vaccaro, Emily E. Diller, Renata Pellegrino Da Silva, Fernanda Mafra, Sha Cao, Katherine B. Stanley, Aaron A. Cohen-Gadol, Jason G. Parker

**Affiliations:** 1 Radiology and Imaging Sciences, School of Medicine, Indiana University, 950 W. Walnut St., R2 E107, Indianapolis, IN 46202, USAparkerjg@iu.edu (J.G.P.); 2 School of Health Sciences, Purdue University, West Lafayette, IN 47907, USA; 3 Center for Applied Genomics, Children’s Hospital of Philadelphia, Philadelphia, PA 19104, USA; 4 Feinberg School of Medicine, Northwestern Medicine, Chicago, IL 60611, USA; 5 10x Genomics, Pleasanton, CA 94588, USA

**Keywords:** multiparametric MRI, intratumoral heterogeneity, machine learning, brain tumor, stereotactic biopsy

## Abstract

Intratumoral heterogeneity (ITH) complicates the diagnosis and treatment of glioma, partly due to the diverse metabolic profiles driven by underlying genomic alterations. While multiparametric imaging enhances the characterization of ITH by capturing both spatial and functional variations, it falls short in directly assessing the metabolic activities that underpin these phenotypic differences. This gap stems from the challenge of integrating easily accessible, colocated pathology and detailed genomic data with metabolic insights. This study presents a multifaceted approach combining stereotactic biopsy with standard clinical open-craniotomy for sample collection, voxel-wise analysis of MR images, regression-based GAM, and whole-exome sequencing. This work aims to demonstrate the potential of machine learning algorithms to predict variations in cellular and molecular tumor characteristics. This retrospective study enrolled ten treatment-naïve patients with radiologically confirmed glioma. Each patient underwent a multiparametric MR scan (T1_W_, T1_W-CE_, T2_W_, T2_W_-FLAIR, DWI) prior to surgery. During standard craniotomy, at least 1 stereotactic biopsy was collected from each patient, with screenshots of the sample locations saved for spatial registration to pre-surgical MR data. Whole-exome sequencing was performed on flash-frozen tumor samples, prioritizing the signatures of five glioma-related genes: IDH1, TP53, EGFR, PIK3CA, and NF1. Regression was implemented with a GAM using a univariate shape function for each predictor. Standard receiver operating characteristic (ROC) analyses were used to evaluate detection, with AUC (area under curve) calculated for each gene target and MR contrast combination. Mean AUC for five gene targets and 31 MR contrast combinations was 0.75 ± 0.11; individual AUCs were as high as 0.96 for both IDH1 and TP53 with T2_W_-FLAIR and ADC, and 0.99 for EGFR with T2_W_ and ADC. These results suggest the possibility of predicting exome-wide mutation events from noninvasive, in vivo imaging by combining stereotactic localization of glioma samples and a semi-parametric deep learning method. The genomic alterations identified, particularly in IDH1, TP53, EGFR, PIK3CA, and NF1, are known to play pivotal roles in metabolic pathways driving glioma heterogeneity. Our methodology, therefore, indirectly sheds light on the metabolic landscape of glioma through the lens of these critical genomic markers, suggesting a complex interplay between tumor genomics and metabolism. This approach holds potential for refining targeted therapy by better addressing the genomic heterogeneity of glioma tumors.

## 1. Introduction

**Figure 1:**
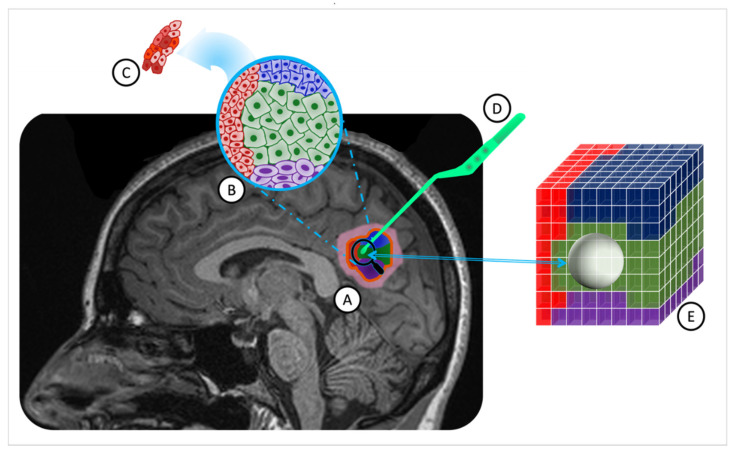
ITH in a solid brain tumor and MRI-guided stereotactic biopsy overlaid on a T1_W_ MR image. (**A**). A solid brain tumor outlined by a yellow border, comprising four distinct cell subpopulations (in red, blue, green, and purple) with associated inflammation in pink. (**B**). Macroheterogeneity within cell subpopulations, displaying reduced cytoplasm and enlarged nuclei in some cells. (**C**). Microheterogeneity among tumor cells of the same subpopulations. (**D**). The pituitary tweezer is used for stereotactic biopsy. (**E**). The tumor sample volume is represented as a sphere, centered on the biopsied location, including two cell populations (blue and green). (ITH: intratumoral heterogeneity; MRI: magnetic resonance imaging; T1_W_: T1-weighted; MR: magnetic resonance).

Increasing mortality rates associated with brain tumors have highlighted a critical need for advancements in both diagnostic and therapeutic approaches [1]. The conventional diagnosis procedure involves pre-surgical imaging and one biopsy sample to assess cellular and molecular properties [2]. This approach enables subsequent optimization of chemotherapy and radiation treatments based on patient-specific mutation profiles. However, the nuanced interplay of genomic alterations and metabolic reprogramming within the tumor microenvironment emerges as a critical consideration. These alterations not only drive genomic instability, a key factor in tumor heterogeneity [3] but also promote distinct metabolic phenotypes within clonal cell populations [4]. Such metabolic shifts, influenced by genetic mutations in key oncogenes and tumor suppressor genes, including IDH1, TP53, EGFR, PIK3CA, and NF1 [5], significantly contribute to ITH by fostering an environment conducive to tumor progression and resistance to therapy [6]. Furthermore, a growing body of both basic and clinical evidence has demonstrated that the somatic and genomic composition of human brain tumors is not uniform across space and time [7]. This phenomenon, referred to as intratumoral heterogeneity (ITH), can compromise the accuracy of results obtained from conventional surgical biopsy, potentially rendering subsequent molecular characterizations incorrect. Though genomic instability is the primary driver of tumor heterogeneity [8], clonal cell subpopulations show plasticity, shifting between cell states [9]. Their proliferation potential is influenced not only by their genetic composition but also by epigenetic factors like DNA methylation and changes in histone structures [10]. ITH can exist as variability in the gene, transcript, or protein levels of distinct cell subpopulations—macroheterogeneity, Figure 1B—or within the cells belonging to the same subpopulation—microheterogeneity [8], Figure 1C. The proliferation capacity of clonal cell subpopulations is modulated not only by their genetic constitution but also by metabolic changes driven by these genetic alterations [4]. Epigenetic alterations, such as DNA methylation and histone modifications, amplify this intricacy by modifying gene expression independently of the changes in the DNA sequence. These epigenetic changes impact tumor cell metabolism, thereby contributing to heterogeneity on both macroscopic and microscopic scales [11]. Therefore, not all the present mutations and expression pathways in the tumor microenvironment (TME) will be identified in the pathology analysis of a conventionally collected single biopsy sample. To overcome this limitation, a noninvasive tool to assess cellular and molecular tissue characteristics across the entire tumor bed and TME is essential.

MRI (magnetic resonance imaging) is the standard presurgical imaging procedure for brain tumors, serving as a noninvasive, multipurpose diagnosis and treatment planning tool [12]. The adoption of MRI was initially driven by its superior contrast resolution in neuroimaging [13]. Now, beyond rendering intricate anatomical and functional insights, it plays a central role in obtaining detailed molecular and cellular characteristics of brain tumors [12]. Combining manifold MRI sequences, known as mpMR (multiparametric magnetic resonance), enables simultaneous assessment of anatomical, functional, and cellular information from the tumor in only one imaging session. A standard brain MRI, with and without contrast, provides morphological and pathophysiological information about the brain tumor, such as edema and necrosis. Integrating MR-derived tumor characteristics with genetic data from biopsy sample analyses using machine learning techniques introduces a promising approach to effectively address the ITH. The foundational hypothesis of this study suggests that, through such integration, a model can be constructed to navigate the heterogeneity challenge, mapping underlying somatic and genomic aberrations with MR imaging signatures.

In a prior retrospective study [14], the potential to predict mutational heterogeneity by utilizing a multiparametric MR-based machine learning algorithm in conjunction with advanced geometric modeling and random field theory was demonstrated. Notably, this initial cohort included a broad spectrum of brain diagnoses, not solely gliomas, and the genetic data was sourced from standard clinical pathology techniques, such as H&E staining and immunohistochemistry, limiting its depth and scope.

The purpose of this current study was to extend our previous work in a population of pure glioma subjects. Data were collected from the routine mpMR imaging acquired as a standard of care for each patient. During surgical intervention, at least one stereotactic tumor biopsy sample was collected prospectively, with the research team meticulously documenting the resection coordinates based on presurgical MR images. Immediately after collection, these samples were flash-frozen in the surgical suite and prepared for subsequent whole exome sequencing (WES). Subsequently, a semiparametric machine learning model was built using the MR signals as predictors and genetic data as outcomes.

In forthcoming sections, we present a detailed methodology offering a potential avenue for understanding the complexities of tumor heterogeneity.

## 2. Methods

### 2.1. Protocol Approval

The Indiana University Institutional Review Board (IRB) approved and monitored this study in accordance with the requirements outlined in US 20 CFR Part 431 [15]. This study presented no more than minimal risk to patients and thus qualified for expedited IRB review under categories two and five that specify “Research involving materials (data, documents, records, or specimens) that have been collected, or will be collected solely for non-research purposes (such as medical treatment or diagnosis). (NOTE: Some research in this category may be exempt from the HHS regulations for the protection of human subjects. 45 CFR 46.101(b)(4)”) [16].

Given that the data collected included protected health information (PHI), appropriate measures were taken to protect subject privacy and confidentiality, including anonymizing all image and clinical data, storing data in secure and redundant institutional storage, and appropriately controlling and restricting access to data. All procedures, methods, and experiments performed in this study were carried out in accordance with relevant guidelines and regulations, including the Declaration of Helsinki and the HIPAA Privacy Rule. This study was not listed on CinicalTrials.gov, and no part of the dataset presented here has been used or published in the past.

### 2.2. Study Population

Ten patients (mean age, 47.0 ± 17.7 years; age range, 25–71 years; seven males and three females) with radiologically diagnosed primary glioma grade II to IV according to the current WHO criteria [17] (5 WHO grade IV, 5 WHO grade II) were included in this study. One patient had a recurring diffuse astrocytoma grade II; all other enrolled subjects were treatment naïve. All subjects enrolled were scheduled for surgical resection of their brain tumor. Each patient had a tumor of sufficient size to guarantee the acquisition of at least one biopsy sample during the surgical procedure. As a part of their standard of care, all patients underwent a clinical multiparametric MR scan within 27.9 ± 34.0 days prior to surgery. Table 1 presents details of the patient cohort. Figure S1 in the Supplementary Materials displays a raincloud plot of the distribution of age, gender, pathological diagnosis, and the range of days between MRI session and surgery, ensuring clear comprehension of the demographic characteristics of the cohort.

### 2.3. Biopsy Sampling and Analysis

Biopsy specimens, with a median size of 4.8 cm^3^, were acquired by the clinical neurosurgeon (ACG) before initiating complete tumor resection. With the patient under general anesthesia and their head stabilized in a three-point head holder, a craniotomy was performed, guided by a frameless stereotactic system. Upon tumor exposure, ACG selected the biopsy location based on areas highlighted by fluorescein fluorescence [18]. Prior to sample collection, the neurosurgeon positioned the pituitary forceps on the target site (Figure 1D). Concurrently, research staff in the operating room captured a screenshot from the stereotactic software (Medtronic Synergy Cranial v2.2.7), ensuring accurate recording of resection coordinates on the presurgical MR images. This method is the least disruptive to the patient’s surgery but is known to be highly operator-dependent. Misregistration errors between the tumor tissue locations in the presurgical MR and the placement of the forceps by the surgeon have been shown in previous studies to result in errors of 2.4 ± 1.7 mm [19].

**Figure 2:**
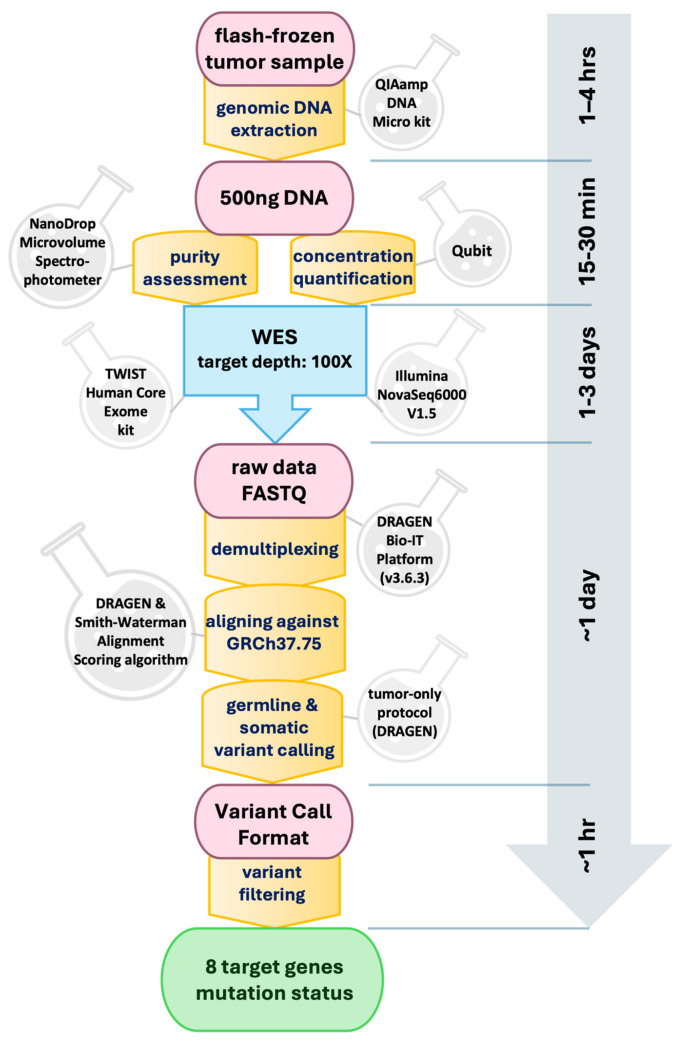
Schematic representation of the biopsy analysis pipeline with whole-exome sequencing (WES) and subsequent bioinformatic analysis. Time estimates for each step are indicated along the vertical axis.

Following collection, each biopsy sample was immediately flash-frozen in the operating room using liquid nitrogen to preserve genetic integrity. Subsequently, genomic DNA extraction was performed from each sample, yielding a minimum of 500 ng of DNA, utilizing the QIAamp DNA Micro Kit (QIAGEN, Germantown, MD, USA). The purity of the extracted DNA was assessed using a NanoDrop Microvolume Spectrophotometer (Thermo Fisher Scientific, Houston, TX, USA), while its concentration was quantified using Qubit. The purified DNA underwent whole-exome sequencing (WES) targeting a depth of 100× by the Center for Applied Genomics (CAG) at CHOP. For sequencing, the Core Exome Capture Kit (TWIST Bioscience, South San Francisco, CA, USA) was employed. Each quality-controlled library was sequenced on an Illumina NovaSeq6000 (V1.5) platform, employing paired-end mode with a read length of 2 × 150 base pairs, achieving an approximate coverage depth of 60× per sample. The acquired data were subsequently demultiplexed using the Illumina DRAGEN Bio-IT Platform (v3.6.3). Alignment of the generated FASTQ files against the Homo sapiens (GRCh37.75) reference was carried out using the DRAGEN pipeline [20], incorporating the Smith-Waterman Alignment Scoring algorithm. Germline variant calling for single-nucleotide variants (SNVs) and insertion/deletion variations were performed, followed by somatic variant calling using a tumor-only protocol within the DRAGEN pipeline, excluding any germline variants. Variant Call Format (VCF) files [21] were then filtered to focus on variants within a predetermined list of eight genes associated with glioma, as detailed in our previous work [5]. Further analysis revealed a significant imbalance in three gene targets—PTEN, PIK3R1, and RB1—with only one positive patient identified for each, as outlined in Table S1 in the Supplementary Materials. The entire process, from DNA extraction to variant filtering, is schematically represented in Figure 2.

### 2.4. MR Data Collection and Analysis

The multiparametric MRI sessions conducted as part of standard care prior to each patient’s biopsy were identified. These sessions typically included at least five common clinical MR contrasts: T1-weighted (T1_W_), T1-weighted with contrast enhancement (T1_W-CE_), T2-weighted (T2_W_), fluid-attenuated inversion recovery (FLAIR) [20,21], and diffusion-weighted imaging (DWI) to produce apparent diffusion coefficient (ADC) maps, providing both anatomical and functional information. These five MR datasets were downloaded from local imaging centers for each patient. Due to clinical protocol variations, two patients were imaged using the T2_W-CE_ sequence in place of T2_W_.

All scans were conducted using a head−neck coil. Imaging for seven patients was performed using a 1.5 T MRI scanner, with scans conducted on five patients using Siemens machines (Siemens Healthineers, Erlangen, Germany) and two patients using GE machines (GE HealthCare, Chicago, IL, USA). Additionally, two patients underwent imaging with a 3.0 T Siemens, and the session for one patient was split between a 1.5 T Siemens scanner and a 3.0 T Toshiba scanner over two consecutive days. Detailed information regarding the types of scanners used and the MR contrast acquired from each subject is provided in Table S2 in the Supplementary Materials.

Quantitative image signals of T1_W_, T2_W_ (or T2_W-CE_), T2_W_-FLAIR, and ADC maps were directly extracted from clinical images. All imaging data were then co-registered to the T1_W-CE_ frame-of-reference with a voxel size of 1 mm^3^. This spatial co-registration process was conducted for each patient using the FMRIB Linear Image Registration Tool (FLIRT) [22,23]. A 12-degree-of-freedom (DOF) cross-correlation objective function was employed for T1_W_ and T2_W_-FLAIR registration [23], while a 12-DOF mutual information objective function for T2_W_ and DWI (only B0) registration [24]. The ADC map for each patient was then aligned with their T1_W-CE_ frame-of-reference using the affine transformation matrix estimated for the DWI B0 images.

Utilizing an automated white matter extraction tool [25,26,27], all images from the five contrasts were normalized to the mean signal of an uninvolved Normal Appearing White Matter (NAWM) region on the T1_W-CE_ image. The volume of tissue noted in the pathology report was used to draw an equal volume sphere on the T1_W-CE_ image, centered on the location where the stereotactic biopsy was marked on three-plane, neuro-navigation MR plans during surgery. This method ensured that the feature matrix and subsequent machine learning model were exclusively limited to the image voxels associated with the resected tissue, as shown in Figure 1E.

### 2.5. Statistical Analysis

To investigate the predictive power of MRI imaging signatures for the mutational status of glioma genomic targets, a generalized additive model (GAM) was developed for regression analysis [28]. GAM, a semi-parametric ensemble machine learning technique, was chosen over less flexible models like generalized linear models (GLMs) better to capture nonlinear and complex relationships [29]. In order to prioritize interpretability, more complex models, such as neural networks, were not chosen. This approach allows for insights into local predictor contributions and ensures transparent results.

**Figure 3:**
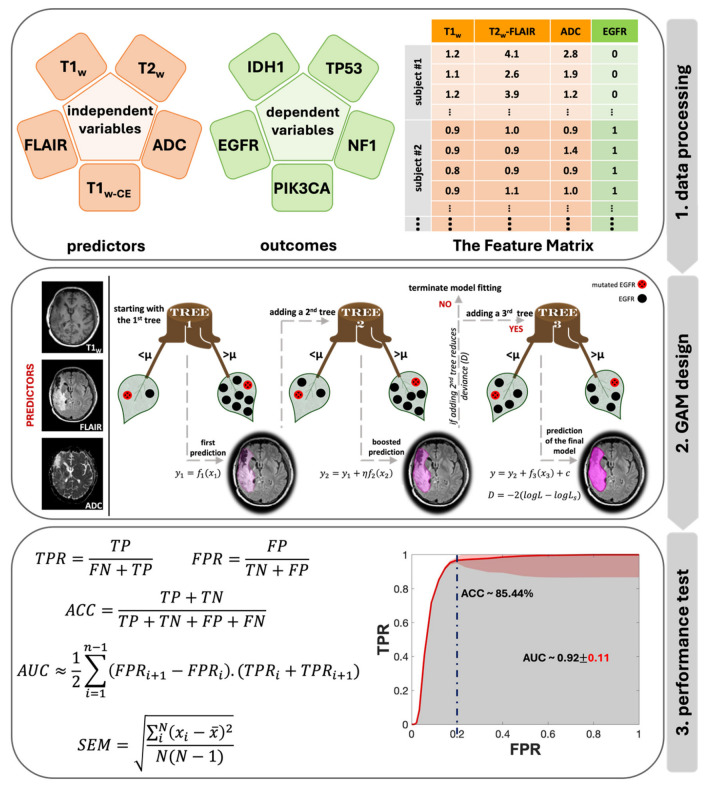
Data analysis flowchart, including the main three steps of the data analysis pipeline: 1. data processing, 2. GAM design with the prediction stages in shades of purple, and 3. performance test with the SEM in red.

Figure 3 displays the flowchart of the data analysis process, beginning with the creation of the feature matrix and ending with testing the model’s performance. In Step 1, a sample feature matrix is formulated comprising mpMR signatures as independent variables and binary class indicators representing the mutation status of target genes as dependent variables for each subject. The data corresponding to each voxel makes up a row of this matrix. Each row of these matrices represents the data corresponding to a voxel. Depending on the volume of the biopsy sample, each subject’s matrices included MR signatures acquired from an average of 10,725 to up to 31,463 voxels per contrast.

To analyze the impact of combined MR contrasts as predictors alongside each contrast separately, we prepared a total of 31 feature matrices categorized into five groups based on the number of contrasts: single, double, triple, quadruple, and quintuple (or all contrasts). Step 2 in Figure 3 provides an architectural visualization of gradient boosting in GAM as a sequential ensemble, using an example of triple contrast (T1_W_, T2_W_-FLAIR, and ADC) for predicting EGFR mutation status. The general form of a GAM can be expressed with a univariate shape function [30]:

(1) $$y=f_{1}(x_{1})+f_{2}\left(x_{2}\right)+\ldots +f_{n}\left(x_{n}\right)+c$$

Here, y is the response variable, $f_{n}\left(x_{n}\right)$ is the univariate shape function (a boosted tree for a linear term for the predictor $x_{n}$), and c is the intercept. The response variable y follows a normal distribution with a mean μ and standard deviation σ. This model was fitted to the dataset using the “fitrgam” function available in the Statistics and Machine Learning Toolbox of MATLAB R2022a (MathWorks, Natick, MA, USA) [31]. This function fits a GAM using a gradient boosting algorithm, which incorporates weak learners, typically presented by decision stumps. At each step, the ensemble fits a new learner to the difference between the observed response and the aggregated prediction of all learners grown previously, aiming to minimize mean-squared error (Step 2 in Figure 3). Deviance, D, is a generalization of the residual sum of squares, is used to measure the goodness of model fit, and is calculated as [32]:

(2) $$D=-2\left(\mathrm{logL}-{\mathrm{logL}}_{s}\right)$$

where L and $L_{s}$ are the likelihoods of the fitted and saturated model, respectively. During its iterations, GAM identifies a learning rate (η) to reduce the deviance (D) for every observed response ($y_{i}$).

We conducted a total of 1550 machine learning experiments, encompassing 31 distinct combinations of five MR contrasts, evaluated against five gene targets for all 10 patients (31 × 5 × 10). For each iteration, the training feature matrix was constructed using both imaging and genetic data from nine patients, reserving the tenth for the test feature matrix. This leave-one-patient-out methodology [33] ensured that every patient, across all 10 individuals, was singularly used as the test subject once, guaranteeing no overlap between training and testing data in any iteration.

For the last step (Step 3 in Figure 3), to test the performance of this model, standard receiver operating characteristic (ROC) analyses were applied to every combination for each genetic target. For each iteration, the true positive rate (TPR), false positive rate (FPR) values and accuracy (ACC) at FPR of 0.2 were calculated using the following equations:

(3) $$\mathrm{TPR}=\frac{\mathrm{True}\;\mathrm{Positives}\;\left(\mathrm{TP}\right)}{\mathrm{False}\;\mathrm{Negatives}\;\left(\mathrm{FN}\right)+\mathrm{True}\;\mathrm{Positives}\;\left(\mathrm{TP}\right)}$$

(4) $$\mathrm{FPR}=\frac{\mathrm{False}\;\mathrm{Positives}\;\left(\mathrm{FP}\right)}{\mathrm{True}\;\mathrm{Negatives}\;\left(\mathrm{TN}\right)+\mathrm{False}\;\mathrm{Positives}\;\left(\mathrm{FP}\right)}$$

(5) $$\mathrm{ACC}=\frac{\mathrm{TP}+\mathrm{TN}}{\mathrm{TP}+\mathrm{TN}+\mathrm{FP}+\mathrm{FN}}$$

To enhance the robustness of the ROC, a maximum threshold of 0.05 was employed, and the standard error of the mean (SEM) was calculated for each threshold using Equation (6):

(6) $$\mathrm{SEM}=\sqrt{\frac{\sum_{i}^{N}{\left(x_{i}-\bar{x}\right)}^{2}}{N\left(N-1\right)}}$$

where N is the number of iterations, $x_{i}$ is the i^th^ measurement and $\bar{x}$ is the mean value of the dataset. The area under the curve (AUC) of each ROC was measured using Equation (7):

(7) $$\mathrm{AUC}\approx \frac{1}{2}\sum_{i=1}^{n-1}({\mathrm{FPR}}_{i+1}-{\mathrm{FPR}}_{i}).\left({\mathrm{TPR}}_{i}+{\mathrm{TPR}}_{i+1}\right)$$

where n is the number of thresholds and $F/{\mathrm{TPR}}_{i}$ and $F/{\mathrm{TPR}}_{i+1}$ are the rates at adjacent thresholds. The SEM was calculated for AUC scores over all the iterations for each unique contrast combination per mutational target. Standard one-way ANOVA (analysis of variance) was performed for AUC comparison using the “anova1” function available in the Statistics and Machine Learning Toolbox of MATLAB R2022a (MathWorks, Natick, MA, USA) [31].

## 3. Results

**Figure 4:**
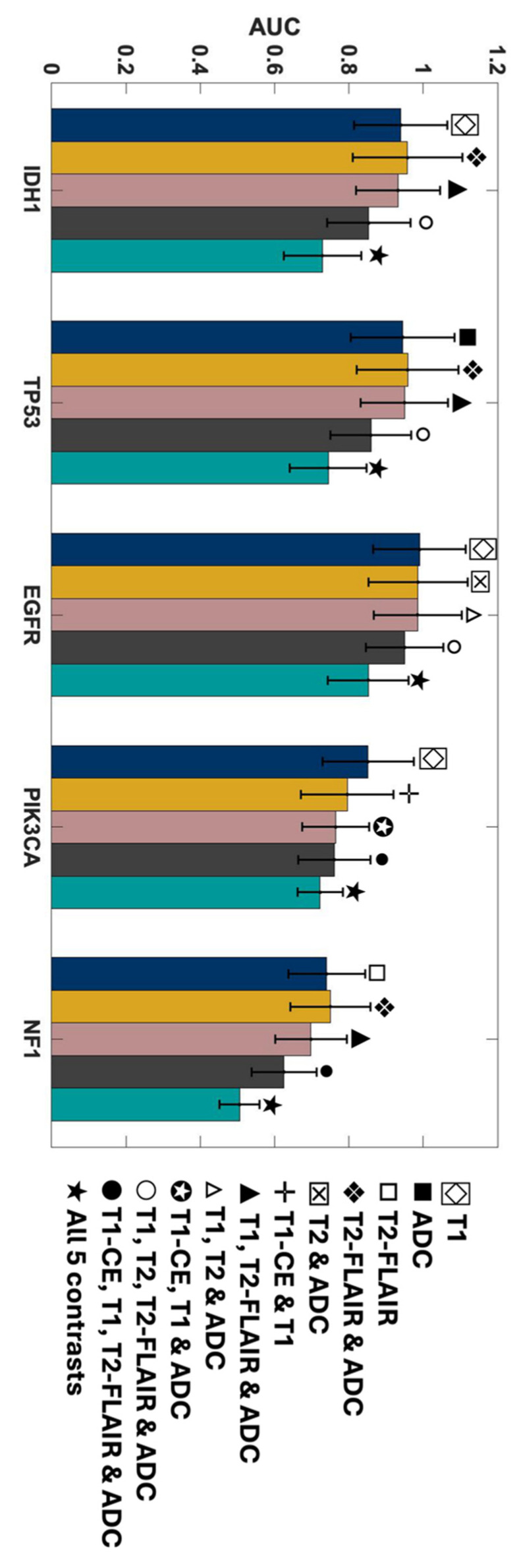
AUC scores of the MRI contrast combinations with the highest mean AUC scores in each group for predicting each mutation status. The SEM bars are indicated on top of each predictor. Navy represents single contrasts, mustard for double contrasts, pink for triple contrasts, smoke for quadruple contrasts, and teal indicates quintuple contrasts.

**Figure 5:**
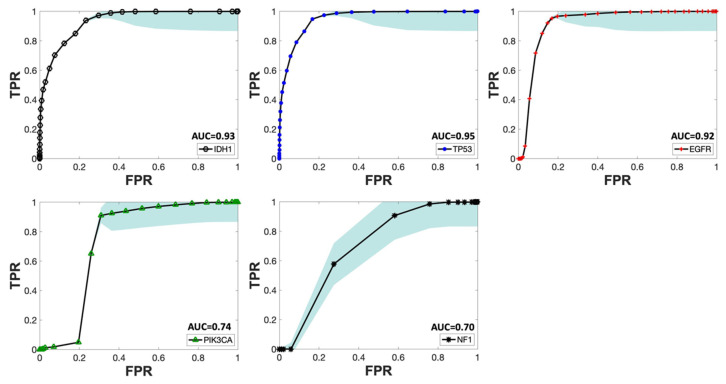
Mean ROC curve of T1_W_, T2_W_-FLAIR, and ADC as predictors for all five mutations across different iterations. The SEM bands for each mutation are depicted in sky blue. The mean AUC score for each curve is indicated next to the predictors’ names in the legend for each mutational target.

**Figure 6:**
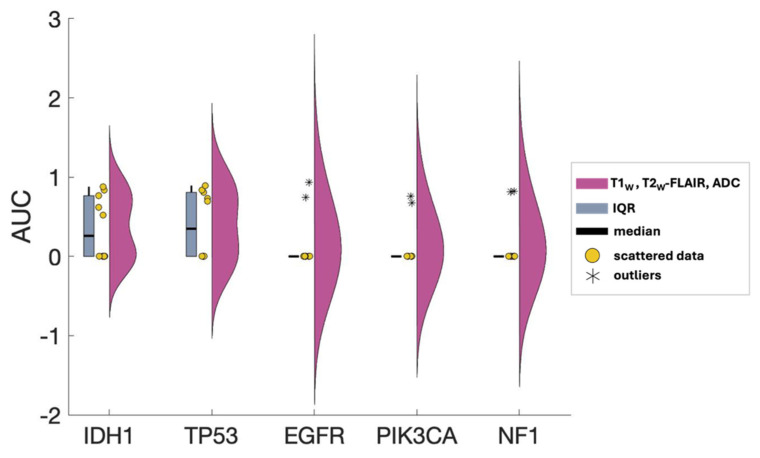
AUC scores distribution of T1_W_, T2_W_-FLAIR, and ADC as predictors for all five mutations. Pink raincloud plots: Data from 10 iterations. Yellow circles: Scattered data. Blue central box: interquartile range (IQR) denoting Q0.25 and Q0.75 quartiles. **Black** line: median. Outliers are shown as asterisks (*) beyond 1.5xIQR.

In this work, the ability of individual MR contrasts to predict genomic features in glioma was assessed by conducting ROC analyses. The observed average ACC values for FPR of 0.2 ranged between 0.71 and 0.83, indicative of statistical robustness. The AUC scores for each of the 31 combinations pertaining to each genetic target are detailed in Figures S2–S6 in the Supplementary Materials. Figure 4 displays the predictor combinations yielding the highest mean AUC scores within each group for each mutation status with their SEM (standard error of the mean) bars indicated. This figure highlights the variations in mean AUC scores upon the inclusion of additional contrasts as predictors. Particularly, T1_W_ and ADC, with T2_W_-FLAIR following closely, were the most frequently occurring MR contrasts within predictor combinations, resulting in higher mean AUC scores for all five mutations. Figure 5 shows the mean ROC curves of the T1_W_, ADC, and T2_W_-FLAIR combination, resulting from averaging over all 10 iterations, along with the SEM band. For the gene targets IDH1, TP53, and EGFR, the AUC scores exceeded 0.9, signifying excellent predictivity. Additionally, the scores for PIK3CA and NF1 registered above 0.7, indicative of acceptable performance. Figure 6 showcases raincloud plots [34] depicting the distribution of AUC scores for all five genes using T1_W_, T2_W_-FLAIR, and ADC imaging signatures as predictors. Each raincloud plot represents the AUC scores obtained from 10 iterations of the predictive modeling process. The central box in each plot signifies the interquartile range (IQR), with the median indicated by the horizontal line. The upper and lower borders of the box correspond to the upper (Q3) and lower (Q1) quartiles, respectively. Outliers are represented as individual points beyond 1.5 times the IQR from the upper (0.75) or lower (0.25) quartiles.

**Figure 7:**
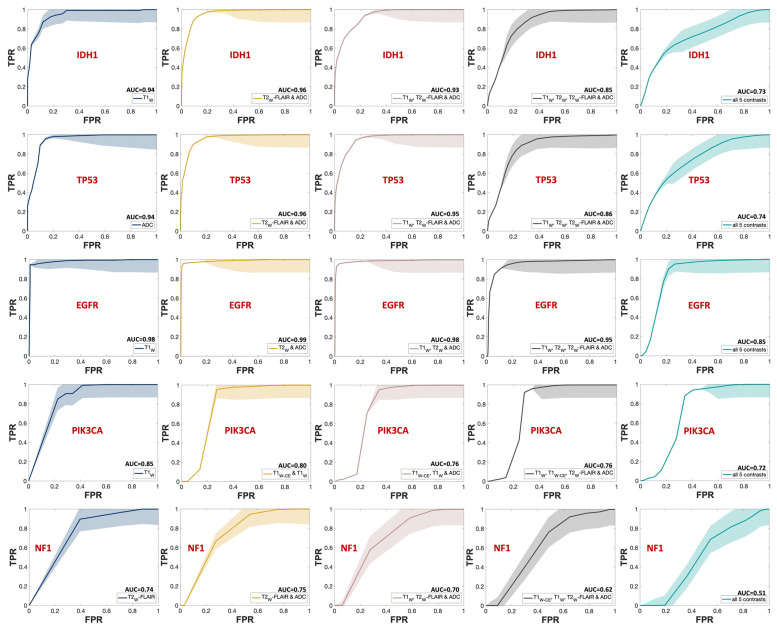
ROC curves of the MRI contrast combinations with the highest mean AUC scores in each group for predicting each mutation status. The SEM uncertainty bands are indicated with the same but lighter color for each graph. Each row is dedicated to one mutational target, starting with IDH1. Navy represents single contrasts, mustard for double contrasts, pink for triple contrasts, smoke for quadruple contrasts, and teal indicates quintuple contrasts. The mean AUC score for each curve is indicated next to the predictors’ names in the legend for each mutational target.

The outstanding performance of the model in predicting IDH1, TP53, and EGFR, achieving a TPR (true positive rate) of 1 with minimal FPR (false positive rate), is evident from the ROC curves shown in Figure 7, corresponding to the same combinations in Figure 4. Each group of triple and quadruple contrasts included T1_W_ and ADC, while double contrast groups featured at least one of them. Among the 20 combinations shown in Figure 7, excluding quintuple contrasts, T1_W_ appeared in 14, ADC in 15, and T2_W_-FLAIR in 12. Integrating all MR contrasts together as independent variables (teal line) did not seem to improve the AUC score in most cases. There was a slight improvement for IDH1 only when compared to using ADC as a predictor by itself. The result of the one-way ANOVA was nonsignificant when comparing AUC scores from different MR combinations, as shown by the p-values presented in Table S3 of the Supplementary Materials.

## 4. Discussion

In this study, a regression-based GAM was developed and tested utilizing clinical MR images to predict the mutational status of select glioma genomic targets. All combinations of T1_W_, T1_W-CE_, T2_W_, T2_W_-FLAIR, and ADC were encompassed in the model training as predictors, with the mutation status of IDH1, TP53, EGFR, PIK3CA, and NF1 as outcomes. The training was conducted over 10 patients using the leave-one-patient-out method. ROC curve analysis was completed for each of the 31 total combinations, and AUC scores were calculated and compared through ANOVA. T1_W_ and ADC emerged as the foremost contrasts, with T2_W_-FLAIR ranking next in higher mean AUC scores for all five targets, resulting in AUC scores as high as 0.98, accompanied by robust ACC values. The ANOVA results were nonsignificant; however, due to the limited dataset, it was not possible to demonstrate the statistical superiority of any of the different combinations as a function of the number of contrasts.

The selected MRI sequences for this study are integral components of the clinical neuroimaging routine for glioma patients. Their incorporation into the clinical practice is not arbitrary but rather grounded in a robust body of literature that emphasizes their clinical relevance. For instance, high-resolution 3D T1_W_ and T2_W_ sequences, along with T1_W-CE_, are essential for detecting abnormalities in the blood-brain barrier (BBB) and areas with increased vascularity [35,36]. The T2_W_-FLAIR technique enhances the visibility of lesions in the periventricular and peripheral subcortical regions by suppressing the CSF signal and reducing the contrast between gray and white matter [37]. This allows for the differentiation of vasogenic edema from normal brain fluids and aids in identifying infiltrative microscopic pathology [38]. The apparent diffusion coefficient (ADC) map, derived from the DWI signal, provides insights into the glioma tumor cellularity [39,40], and its utility in grading these tumors has been well studied [41,42,43].

As shown in Figure 4 and Figure 7, the mean AUC score for T2_W_ contrast in the single contrast group was never the highest for any genetic targets. The T2_W_ signal’s contribution was better seen when combined with other contrasts. This outcome is somewhat unexpected since T2_W_ images are routinely used in clinical brain tumor diagnosis [44]. However, their primary application in such contexts is for qualitative visual assessment, not the signal quantification that was employed in building this model. One explanation for this outcome could be the substitution of T2_W_ with Gadolinium-enhanced T2_W_ (T2_W-CE_) images for two subjects in the training (Table S2 in the Supplementary Materials). This change was implemented because two patients lacked T2_W_ images in their most recent presurgical imaging dataset and were instead scanned with T2_W-CE_ sequences. In addition, while Gadolinium-based contrast agents can diminish the T2_W_ signal, a notable decrease [45] only occurs when the administered dose is higher than the FDA-approved limit (1 mmol/kg) [46,47], which was not the case in this study.

Gliomas exhibit a spectrum of genetic alterations that vary depending on their grade and specific subtype [48], with significant prognostic implications extensively studied [49]. IDH1 mutations, producing the oncometabolite 2-HG that disrupts cell differentiation and influences tumor behavior, are commonly found in lower-grade gliomas and certain glioblastomas [50], reflecting their distinct metabolic profile. Alterations in EGFR and mutations in TP53, which modulate glucose uptake and metabolism and disrupt glycolysis and oxidative phosphorylation, span various glioma grades [51,52,53]. This study obtained excellent AUC scores for IDH1, TP53, and EGFR, aligning with existing literature and underscoring the interplay between these genetic markers and metabolic pathways in glioma heterogeneity. Figure 5 presents the ROC curves for these three markers, using T1_W_, ADC, and T2_W_-FLAIR MR contrast combination. The distribution of AUC scores for each of these ROC curves over all 10 iterations is depicted in Figure 6. Given that the WES analysis on most of the subjects’ tumor samples yielded no mutations for EGFR, PIK3CA, and NF1 (Table S2 in the Supplementary Materials), the kernel density distribution is more concentrated near the median (0). This underscores the potential benefits of a larger number of biopsy samples to balance the extensive imaging data and enhance the reliability and robustness of the model’s predictive abilities.

Additionally, mutations in PIK3CA and NF1—associated with alterations in lipid metabolism and the activation of glycolysis through the RAS signaling pathway, respectively—are frequently linked to glioblastoma, glioma WHO grade IV [54,55] but rare in lower-grade gliomas except for NF1 alterations in pediatric optic nerve gliomas [56]. The calculated AUC score for PIK3CA and NF1 as dependent variables was near 0.7, as shown in Figure 4, Figure 5 and Figure 7. This comparatively weaker predictive power, consistent with the literature, takes into account that only half of our cohort had a glioblastoma diagnosis, without data stratification by glioma grade during model construction, reflecting the nuanced relationship between genetic alterations, their metabolic consequences, and tumor heterogeneity [48,49,50,51,52,53,54,55,56].

Another important observation of this study is that the combination of all MR contrasts together as predicting variables (teal bar in Figure 4 and teal line in Figure 7) did not enhance the AUC score compared to most of the combinations of fewer contrasts. Although assessing the underlying mechanism of this pattern is challenging, it is well-known that adding independent variables increases model sparseness and may lead to feature redundancy [57]. With a limited sample size, including five distinct MR contrasts increases the risk of introducing numerous features, some of which may be redundant or conflicting, complicating the algorithm’s ability to discern relevant patterns. This comparison should be revisited with a larger cohort or, at the very least, a greater number of biopsy samples per subject.

### Limitations

While this study yields promising results, it is important to acknowledge several limitations. Firstly, the relatively small cohort of 10 subjects used for model training may limit the generalizability of findings (Figure S1 in the Supplementary Materials). In addition, this limitation hinders the ability to demonstrate the effectiveness of the different combinations based on the number of contrasts. At an individual patient level, most datasets were overpowered, with an average of 10,736 voxels per MR contrast per subject, thus yielding statistically significant findings. However, we anticipate that these models may lack generalizability due to the limited number of patients, which does not adequately represent the large and heterogenous brain tumor population. Therefore, a larger sample size would likely enhance the robustness and accuracy of the model. Secondly, the MRI data were obtained as part of the standard clinical routine, leading to variability in imaging parameters across subjects and imaging facilities and decreased harmonization (Table S2 in Supplementary Materials). This variability could introduce inconsistencies in certain MRI sequence parameters, potentially impacting model performance. Thirdly, the reliance on genomic information obtained from a single stereotactic biopsy sample per patient may not fully capture intra-tumoral heterogeneity. Additionally, our choice of the generalized additive model (GAM) was deliberate, as it offered a balance between flexibility and interpretability. Its semiparametric design allowed for capturing complex correlations and interactions in the data without imposing strict assumptions on the underlying relationships. However, it is important to acknowledge that the selection of the machine learning method can significantly impact prediction results, such as leading to problems like overfitting [58]. While GAM proved effective for our analysis, alternative methods may uncover different underlying mechanisms. Future studies could explore and compare alternative machine learning approaches to gain further insights into the predictive modeling of glioma genomic targets. Lastly, our analysis focused on five main mutation targets as outcome variables. Future research should explore other potential genomic markers associated with glioma to provide a more comprehensive assessment of glioma genetics and elevate the clinical utility of these models.

## 5. Conclusions

This study contributes to the body of evidence integrating MRI imaging and stereotactic biopsy sampling with advanced statistical modeling to predict glioma genomic targets accurately. This type of experimental design is challenging to administer but provides robust and highly controlled data collection on intra-tumoral heterogeneity, the driving factor in human brain tumor treatment resistance and recurrence. The identified genomic alterations, notably in IDH1, TP53, EGFR, PIK3CA, and NF1, are recognized for their significant involvement in metabolic pathways that contribute to glioma heterogeneity. Consequently, our methodology offers insight into the metabolic dynamics of glioma by examining these crucial genomic markers, unveiling the intricate interplay between tumor genomics and metabolism. The regression-based GAM presented here exhibits remarkable promise in using T1_W_, ADC, and T2_W_-FLAIR, especially for key mutations like IDH1, TP53, and EGFR. However, certain nuances, such as the predictability for markers such as PIK3CA and NF1, suggest the need for further refinement and larger cohorts for improved accuracy. An important future direction of this work will involve conducting multisite trials to ensure sufficient statistical sampling for identifying the optimal contrast combinations. This study lays a foundation for future work in noninvasive tumor diagnostics, enhancing treatment precision while minimizing patient risk. As the medical community gravitates towards individualized treatment plans, such innovative approaches will be instrumental in revolutionizing patient care in neuro-oncology.

## Figures and Tables

**Table 1:** Patient demographics and cohort.

Total patients	10	Radiologic diagnosis	10 WHO grade II-IV glioma
Male	7	Pathologic diagnosis	5 glioblastoma, 2 oligodendroglioma (grade II), 3 astrocytoma (grade II)
Age (mean ± STDV)	47.0 ± 17.7 years	$\bar{\Delta_{SX-IMG}}$ (mean ± STDV)	27.9 ± 34.0 days
Age range	25–71 years	$R_{SX-IMG}$	0–96 days
Oncologic status	10 primary	Number of samples (mean ± STDV)	1.8 ± 0.4

$\bar{\Delta_{SX-IMG}}$: The average days between MRI session and surgery for all patients. $R_{SX-IMG}$: The range of days between MRI session and surgery for all patients. (STDV: standard deviation; WHO: world health organization).

## Data Availability

The original contributions presented in the study are included in the article and the Supplementary Materials. Further inquiries can be directed to the corresponding author.

## Supplementary Materials

The following supporting information can be downloaded at https://www.mdpi.com/article/10.3390/metabo14060337/s1, Table S1. Mutation status of 8 glioma targets for 10 subjects, where “1” denotes mutated and “0” signifies no mutation. The table highlights the imbalance observed in PTEN, PIK3R1, and RB1, with only one patient exhibiting mutations, depicted by cells shaded in orange. Table S2. Summary of MR scanner types, MR contrasts, pathological diagnosis, and WES results per patient. Inconsistencies in MRI contrasts or major MR Scanner (*) differences highlighted in orange. Table S3. The p-values from one-way ANOVA for all 5 mutational targets. Rows 1 to 4 display the p-value from ANOVA on AUC scores from different groups of MR contrasts combinations (single, double, triple, quadruple). The p-values in the final row are derived from one-way ANOVA for each mutation across all 31 combinations. Figure S1. Raincloud distribution of cohort demographic characteristics: age, gender, pathological diagnosis based on WHO, and the number of days between MRI session and surgery (R_SX-IMG_). Yellow circles: Scattered data. Blue central box: interquartile range (IQR) denoting Q0.25 and Q0.75 quartiles. Black line: median. Outliers shown as asterisks (*) beyond 1.5xIQR. Figure S2. AUC scores of different MRI contrast combinations for predicting IDH1 mutation status with SEM error bars. Figure S3. AUC scores of different MRI contrast combinations for predicting TP53 mutation status with SEM error bars. Figure S4. AUC scores of different MRI contrast combinations for predicting EGFR mutation status with SEM error bars. Figure S5. AUC scores of different MRI contrast combinations for predicting PIK3CA mutation status with SEM error bars. Figure S6. AUC scores of different MRI contrast combinations for predicting NF1 mutation status with SEM error bars.

## References

[1] Miller K.D., Ostrom Q.T., Kruchko C., Patil N., Tihan T., Cioffi G., Fuchs H.E., Waite K.A., Jemal A., Siegel R.L. (2021). Brain and other central nervous system tumor statistics, 2021. CA Cancer J. Clin..

[2] Mesfin F.B., Al-Dhahir M.A. (2017). Gliomas.

[3] Raynaud F., Mina M., Tavernari D., Ciriello G. (2018). Pan-cancer inference of intra-tumor heterogeneity reveals associations with different forms of genomic instability. PLoS Genet..

[4] Seth Nanda C., Venkateswaran S.V., Patani N., Yuneva M. (2020). Defining a metabolic landscape of tumours: Genome meets metabolism. Br. J. Cancer.

[5] Zhang M., Yang D., Gold B. (2019). Origin of mutations in genes associated with human glioblastoma multiform cancer: Random polymerase errors versus deamination. Heliyon.

[6] Lang F., Liu Y., Chou F.-J., Yang C. (2021). Genotoxic therapy and resistance mechanism in gliomas. Pharmacol. Ther..

[7] Vitale I., Shema E., Loi S., Galluzzi L. (2021). Intratumoral heterogeneity in cancer progression and response to immunotherapy. Nat. Med..

[8] Kashyap A., Rapsomaniki M.A., Barros V., Fomitcheva-Khartchenko A., Martinelli A.L., Rodriguez A.F., Gabrani M., Rosen-Zvi M., Kaigala G. (2022). Quantification of tumor heterogeneity: From data acquisition to metric generation. Trends Biotechnol..

[9] Yabo Y.A., Niclou S.P., Golebiewska A. (2022). Cancer cell heterogeneity and plasticity: A paradigm shift in glioblastoma. Neuro-oncology.

[10] Kreso A., O’Brien C.A., Van Galen P., Gan O.I., Notta F., Brown A.M., Ng K., Ma J., Wienholds E., Dunant C. (2013). Variable clonal repopulation dynamics influence chemotherapy response in colorectal cancer. Science.

[11] Handy D.E., Castro R., Loscalzo J. (2011). Epigenetic modifications: Basic mechanisms and role in cardiovascular disease. Circulation.

[12] DeAngelis L.M. (2001). Brain tumors. N. Engl. J. Med..

[13] Liang Z.-P., Lauterbur P.C. (2000). Principles of Magnetic Resonance Imaging.

[14] Parker J.G., Diller E.E., Cao S., Nelson J.T., Yeom K., Ho C., Lober R. (2019). Statistical multiscale mapping of IDH1, MGMT, and microvascular proliferation in human brain tumors from multiparametric MR and spatially-registered core biopsy. Sci. Rep..

[15] Edwards U. (2010). 10 CFR Part 431. Fed. Regist..

[16] (2009). Code of Federal Regulations Title 45 Public Welfare Part 46 Protection of Human Subjects.

[17] Osborn A., Louis D., Poussaint T., Linscott L., Salzman K. (2022). The 2021 World Health Organization classification of tumors of the central nervous system: What neuroradiologists need to know. Am. J. Neuroradiol..

[18] Li Y., Rey-Dios R., Roberts D.W., Valdés P.A., Cohen-Gadol A.A. (2014). Intraoperative fluorescence-guided resection of high-grade gliomas: A comparison of the present techniques and evolution of future strategies. World Neurosurg..

[19] Sarkiss C.A., Rasouli J.J., Hadjipanayis C.G. (2016). Intraoperative imaging of glioblastoma. Glioblastoma E-Book.

[20] Zhao S., Agafonov O., Azab A., Stokowy T., Hovig E. (2020). Accuracy and efficiency of germline variant calling pipelines for human genome data. Sci. Rep..

[21] Danecek P., Auton A., Abecasis G., Albers C.A., Banks E., DePristo M.A., Handsaker R.E., Lunter G., Marth G.T., Sherry S.T. (2011). The variant call format and VCFtools. Bioinformatics.

[22] Jenkinson M., Smith S. (2001). A global optimisation method for robust affine registration of brain images. Med. Image Anal..

[23] Jenkinson M., Bannister P., Brady M., Smith S. (2002). Improved optimization for the robust and accurate linear registration and motion correction of brain images. Neuroimage.

[24] Greve D.N., Fischl B. (2009). Accurate and robust brain image alignment using boundary-based registration. Neuroimage.

[25] Jenkinson M., Beckmann C.F., Behrens T.E., Woolrich M.W., Smith S.M. (2012). Fsl. Neuroimage.

[26] Sun X., Shi L., Luo Y., Yang W., Li H., Liang P., Li K., Mok V.C., Chu W.C., Wang D. (2015). Histogram-based normalization technique on human brain magnetic resonance images from different acquisitions. Biomed. Eng. Online.

[27] Roy S., Carass A., Prince J.L. Patch based intensity normalization of brain MR images. Proceedings of the 2013 IEEE 10th International Symposium on Biomedical Imaging.

[28] Hastie T.J. (2017). Generalized additive models. Statistical Models in S.

[29] Lou Y., Caruana R., Gehrke J., Hooker G. Accurate intelligible models with pairwise interactions. Proceedings of the 19th ACM SIGKDD International Conference on Knowledge Discovery and Data Mining.

[30] Lou Y., Caruana R., Gehrke J. Intelligible models for classification and regression. Proceedings of the 18th ACM SIGKDD International Conference on Knowledge Discovery and Data Mining.

[31] (2022). MATLAB.

[32] Zuur A.F., Ieno E.N., Walker N., Saveliev A.A., Smith G.M., Zuur A.F., Ieno E.N., Walker N.J., Saveliev A.A., Smith G.M. (2009). GLM and GAM for count data. Mixed Effects Models and Extensions in Ecology with R.

[33] Hastie T., Tibshirani R., Friedman J.H., Friedman J.H. (2009). The Elements of Statistical Learning: Data Mining, Inference, and Prediction.

[34] Karvelis P. (2024). Daviolinplot—Beautiful Violin and Raincloud Plots.

[35] Lee E.K., Lee E.J., Kim S., Lee Y.S. (2016). Importance of contrast-enhanced fluid-attenuated inversion recovery magnetic resonance imaging in various intracranial pathologic conditions. Korean J. Radiol..

[36] Wen P.Y., Macdonald D.R., Reardon D.A., Cloughesy T.F., Sorensen A.G., Galanis E., DeGroot J., Wick W., Gilbert M.R., Lassman A.B. (2010). Updated response assessment criteria for high-grade gliomas: Response assessment in neuro-oncology working group. J. Clin. Oncol..

[37] Bydder G.M., Young I.R. (1985). MR imaging: Clinical use of the inversion recovery sequence. J. Comput. Assist. Tomogr..

[38] Donahue M.J., Blakeley J.O., Zhou J., Pomper M.G., Laterra J., Van Zijl P.C. (2008). Evaluation of human brain tumor heterogeneity using multiple T1-based MRI signal weighting approaches. Magn. Reson. Med. Off. J. Int. Soc. Magn. Reson. Med..

[39] Sugahara T., Korogi Y., Kochi M., Ikushima I., Shigematu Y., Hirai T., Okuda T., Liang L., Ge Y., Komohara Y. (1999). Usefulness of diffusion-weighted MRI with echo-planar technique in the evaluation of cellularity in gliomas. J. Magn. Reson. Imaging Off. J. Int. Soc. Magn. Reson. Med..

[40] Ellingson B.M., Malkin M.G., Rand S.D., Connelly J.M., Quinsey C., LaViolette P.S., Bedekar D.P., Schmainda K.M. (2010). Validation of functional diffusion maps (fDMs) as a biomarker for human glioma cellularity. J. Magn. Reson. Imaging.

[41] Van Cauter S., Veraart J., Sijbers J., Peeters R.R., Himmelreich U., De Keyzer F., Van Gool S.W., Van Calenbergh F., De Vleeschouwer S., Van Hecke W. (2012). Gliomas: Diffusion kurtosis MR imaging in grading. Radiology.

[42] Lam W., Poon W., Metreweli C. (2002). Diffusion MR imaging in glioma: Does it have any role in the pre-operation determination of grading of glioma?. Clin. Radiol..

[43] Chang P., Malone H., Bowden S., Chow D., Gill B., Ung T., Samanamud J., Englander Z., Sonabend A., Sheth S. (2017). A multiparametric model for mapping cellularity in glioblastoma using radiographically localized biopsies. Am. J. Neuroradiol..

[44] Young G.S. (2007). Advanced MRI of adult brain tumors. Neurol. Clin..

[45] Lee M.-J., Kim M.-J., Yoon C.-S., Song S.Y., Park K., Kim W.S. (2011). The T2-shortening effect of gadolinium and the optimal conditions for maximizing the CNR for evaluating the biliary system: A phantom study. Korean J. Radiol..

[46] Ibrahim M.A., Hazhirkarzar B., Dublin A.B. (2018). Gadolinium Magnetic Resonance Imaging.

[47] Kuperman V.Y., Alley M.T. (1999). Differentiation between the effects of T1 and T2* shortening in contrast-enhanced MRI of the breast. J. Magn. Reson. Imaging Off. J. Int. Soc. Magn. Reson. Med..

[48] Walker C., Baborie A., Crooks D., Wilkins S., Jenkinson M. (2011). Biology, genetics and imaging of glial cell tumours. Br. J. Radiol..

[49] Luo Y., Hou W.-T., Zeng L., Li Z.-P., Ge W., Yi C., Kang J.-P., Li W., Wang F., Wu D.-B. (2020). Progress in the study of markers related to glioma prognosis. Eur. Rev. Med. Pharmacol. Sci..

[50] Yan H., Parsons D.W., Jin G., McLendon R., Rasheed B.A., Yuan W., Kos I., Batinic-Haberle I., Jones S., Riggins G.J. (2009). IDH1 and IDH2 mutations in gliomas. N. Engl. J. Med..

[51] Saadeh F.S., Mahfouz R., Assi H.I. (2018). EGFR as a clinical marker in glioblastomas and other gliomas. Int. J. Biol. Markers.

[52] Furnari F.B., Fenton T., Bachoo R.M., Mukasa A., Stommel J.M., Stegh A., Hahn W.C., Ligon K.L., Louis D.N., Brennan C. (2007). Malignant astrocytic glioma: Genetics, biology, and paths to treatment. Genes Dev..

[53] Noor H., Briggs N.E., McDonald K.L., Holst J., Vittorio O. (2021). Tp53 mutation is a prognostic factor in lower grade glioma and may influence chemotherapy efficacy. Cancers.

[54] Verhaak R.G., Hoadley K.A., Purdom E., Wang V., Qi Y., Wilkerson M.D., Miller C.R., Ding L., Golub T., Mesirov J.P. (2010). Integrated genomic analysis identifies clinically relevant subtypes of glioblastoma characterized by abnormalities in PDGFRA, IDH1, EGFR, and NF1. Cancer Cell.

[55] Gallia G.L., Rand V., Siu I.-M., Eberhart C.G., James C.D., Marie S.K., Oba-Shinjo S.M., Carlotti C.G., Caballero O.L., Simpson A.J. (2006). PIK3CA gene mutations in pediatric and adult glioblastoma multiforme. Mol. Cancer Res..

[56] Campen C.J., Gutmann D.H. (2018). Optic pathway gliomas in neurofibromatosis type 1. J. Child Neurol..

[57] Khalid S., Khalil T., Nasreen S. A survey of feature selection and feature extraction techniques in machine learning. Proceedings of the 2014 Science and Information Conference.

[58] Wood S.N. (2008). Fast stable direct fitting and smoothness selection for generalized additive models. J. R. Stat. Soc. Ser. B Stat. Methodol..

