# Supplementary material for: Metabolic Insight into Glioma Heterogeneity: Mapping Whole Exome Sequencing to In Vivo Imaging with Stereotactic Localization and Deep Learning
Source: Metabolites. 2024 Jun 16;14(6):337. doi: 10.3390/metabo14060337 (PMC11205750; doi:10.3390/metabo14060337)
Supplement: Supplementary file 1 [file metabolites-14-00337-s001.zip › metabolites-2921071-supplementary.pdf]

Supplementary Materials

Metabolic Insight into Glioma Heterogeneity: Mapping Whole Exome Sequencing to In Vivo Imaging with Stereotactic Localization and Deep Learning

Mahsa Servati [1,2,*], Courtney N. Vaccaro [3], Emily E. Diller [4], Renata Pellegrino Da Silva [3], Fernanda Mafra [5], Sha Cao [1], Katherine B. Stanley [1], Aaron A. Cohen-Gadol [1] and Jason G. Parker [1,2]

[1] Radiology and Imaging Sciences, School of Medicine, Indiana University, 950 W. Walnut St., R2 E107, Indianapolis, IN 46202, USA
[2] School of Health Sciences, Purdue University, West Lafayette, IN 47907, USA
[3] Center for Applied Genomics, Children's Hospital of Philadelphia, Philadelphia, PA 19104, USA
[4] Feinberg School of Medicine, Northwestern Medicine, Chicago, IL 60611, USA
[5] 10x Genomics, Pleasanton, CA 94588, USA
* Correspondence: mservati@iu.edu; Tel.: +1-(317)-278-3384; Fax: +1-(317)-274-1067

Subjects	PTEN	IDH1	TP53	EGFR	PIK3R1	PIK3CA	NF1	RB1
patient-01	0	1	1	0	0	0	0	0
patient-02	0	0	0	0	0	0	1	0
patient-03	0	1	1	0	0	0	0	0
patient-04	0	0	1	0	0	0	0	0
patient-05	1	0	0	1	1	1	0	0
patient-06	0	0	0	0	0	0	0	0
patient-07	0	1	1	0	0	1	0	0
patient-08	0	0	0	1	0	0	0	0
patient-09	0	1	1	0	0	0	0	0
patient-10	0	1	1	0	0	0	1	1

Table S1. Mutation status of 8 glioma targets for 10 subjects, where "1" denotes mutated and "0" signifies no mutation. The table highlights the imbalance observed in PTEN, PIK3R1, and RB1, with only one patient exhibiting mutations, depicted by cells shaded in orange.

Subjects	MR Scanner	MR Sequences	Pathological Diagnosis	WES Results
patient-01	1.5T GE, SIGNA Explorer	$T1_W$, $T1_{W-CE}$, $T2_W$, $T2_W$-FLAIR, ADC	Oligodendroglioma, grade *II*	IDH1+, TP53+
patient-02	1.5T Siemens, Amira	$T1_W$, $T1_{W-CE}$, $T2_W$, $T2_W$-FLAIR, ADC	Glioblastoma, grade *IV*	NF1+
patient-03	1.5T Siemens, Aera	$T1_W$, $T1_{W-CE}$, $T2_W$, $T2_W$-FLAIR, ADC	Astrocytoma, grade *II*	IDH1+, TP53+
patient-04	1.5T Siemens, Aera	$T1_W$, $T1_{W-CE}$, $T2_W$, $T2_W$-FLAIR, ADC	Pleomorphic xanthoastrocytoma, grade *II*	TP53+
patient-05	3.0T Siemens, Skyra	$T1_W$, $T1_{W-CE}$, $T2_W$, $T2_W$-FLAIR, ADC	Glioblastoma, grade *IV*	PTEN+, EGFR+, PIK3R1+, PIK3CA+
patient-06	1.5T Siemens, Symphony	$T1_W$, $T1_{W-CE}$, $T2_W$, $T2_W$-FLAIR, ADC	Glioblastoma, grade *IV*	no mutated target
patient-07	3.0T Siemens, Skyra	$T1_W$, $T1_{W-CE}$, $T2_W$, $T2_W$-FLAIR, ADC	Diffuse glioma, grade *II*	IDH1+, TP53+, PIK3CA+
patient-08	1.5T Siemens, Aera	$T1_W$, $T1_{W-CE}$, $T2_{W-CE}$, $T2_W$-FLAIR, ADC	Glioblastoma, grade *IV*	EGFR+
patient-09	1.5T Siemens, Aera & 3.0T Toshiba, Titan*	$T1_W$, $T1_{W-CE}$, $T2_{W-CE}$, $T2_W$-FLAIR, ADC	Glioblastoma, grade *IV*	IDH1+, TP53+
patient-10	1.5T GE, Optima 450W	$T1_W$-FLAIR, , $T1_{W-CE}$, $T2_W$, $T2_W$-FLAIR, ADC	Oligodendroglioma, grade *II*	IDH1+, TP53+, NF1, RB1+

Table S2. Summary of MR scanner types, MR contrasts, pathological diagnosis, and WES results per patient. Inconsistencies in MRI contrasts or major MR Scanner (*) differences highlighted in orange.

	IDH1	TP53	EGFR	PIK3CA	NF1
single contrast groups	0.46	0.42	*1.00*	1.00	0.99
double contrast groups	0.79	0.74	*1.00*	1.00	0.97
triple contrast groups	0.88	0.81	1.00	1.00	0.98
quadruple contrast groups	0.90	0.86	1.00	1.00	0.89
all 31 groups	0.98	0.95	1.00	1.00	1.00

Table S3. The p-values from one-way ANOVA for all 5 mutational targets. Rows 1 to 4 display the p-value from ANOVA on AUC scores from different groups of MR contrasts combinations (single, double, triple, quadruple). The p-values in the final row are derived from one-way ANOVA for each mutation across all 31 combinations.

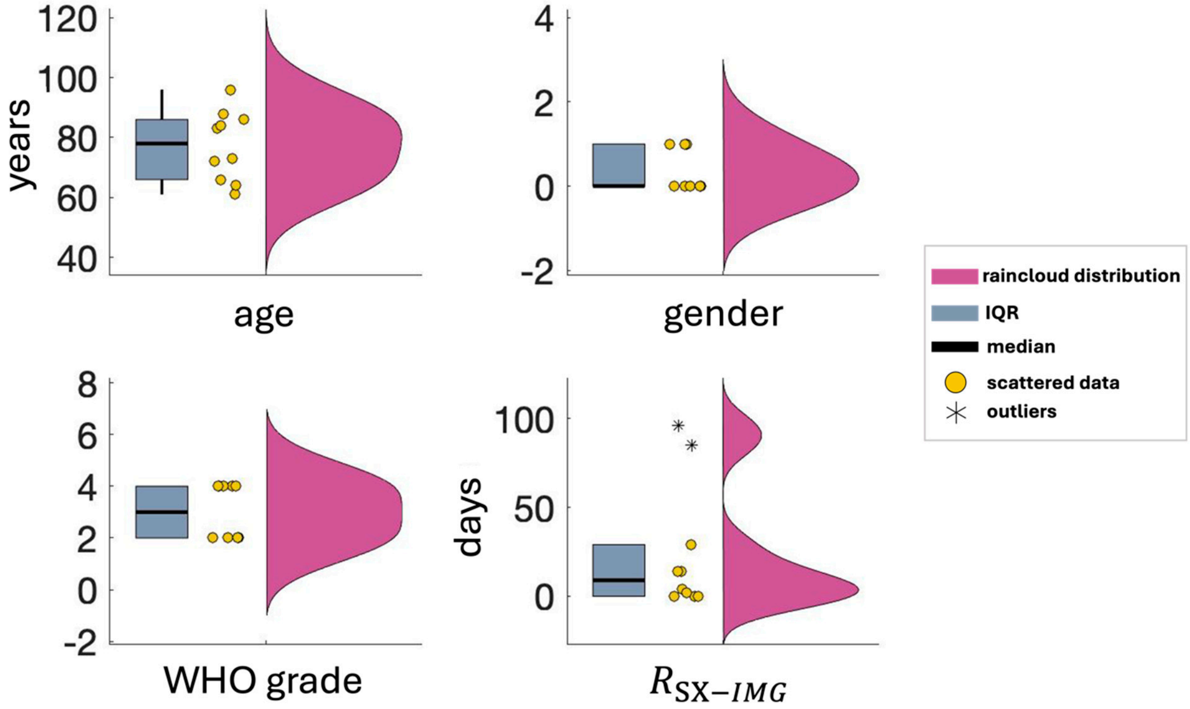

Figure S1. Raincloud distribution of cohort demographic characteristics: age, gender, pathological diagnosis based on WHO, and the number of days between MRI session and surgery ($R_{SX\text{-}IMG}$). Yellow circles: Scattered data. Blue central box: interquartile range (IQR) denoting Q0.25 and Q0.75 quartiles. **Black** line: median. Outliers shown as asterisks (*) beyond 1.5xIQR.

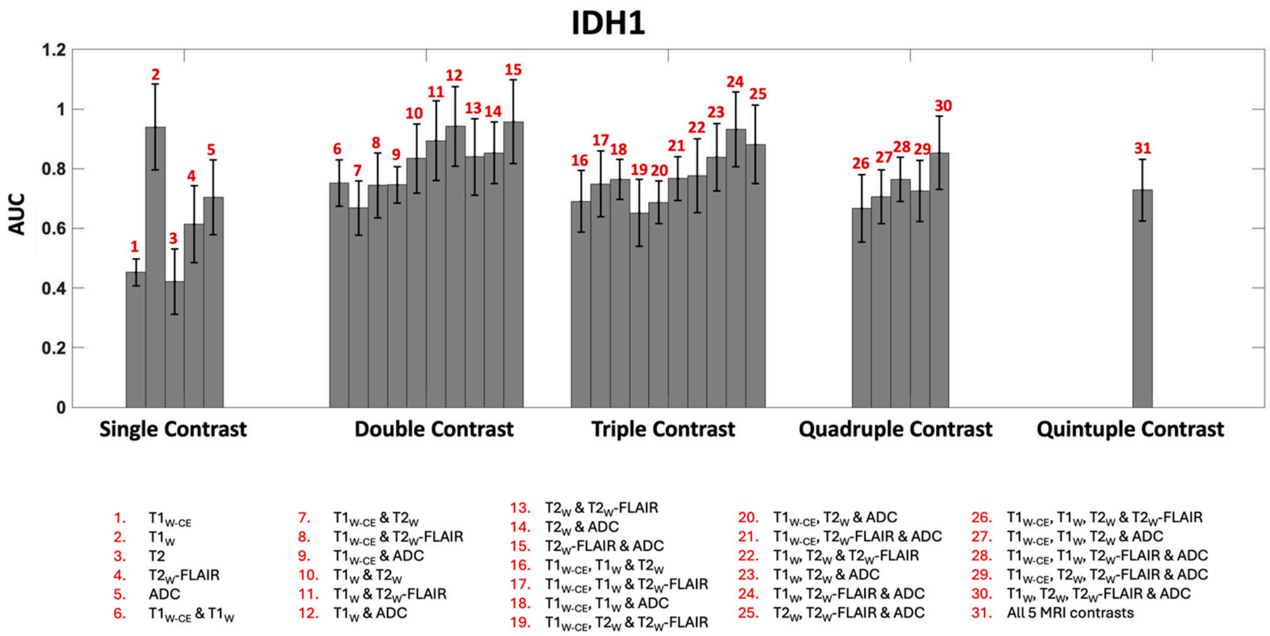

Figure S2. AUC scores of different MRI contrast combinations for predicting IDH1 mutation status with SEM error bars.
(AUC: area under curve; MRI: magnetic resonance imaging; IDH1: isocitrate dehydrogenase 1; $T1_{W-CE}$: contrast enhanced T1-weighted; $T1_W$: T1-weighted; $T2_W$: T2-weighted; $T2_W$-FLAIR: T2-weighted fluid attenuated inversion recovery; ADC: apparent diffusion coefficient, SEM: standard mean of the error).

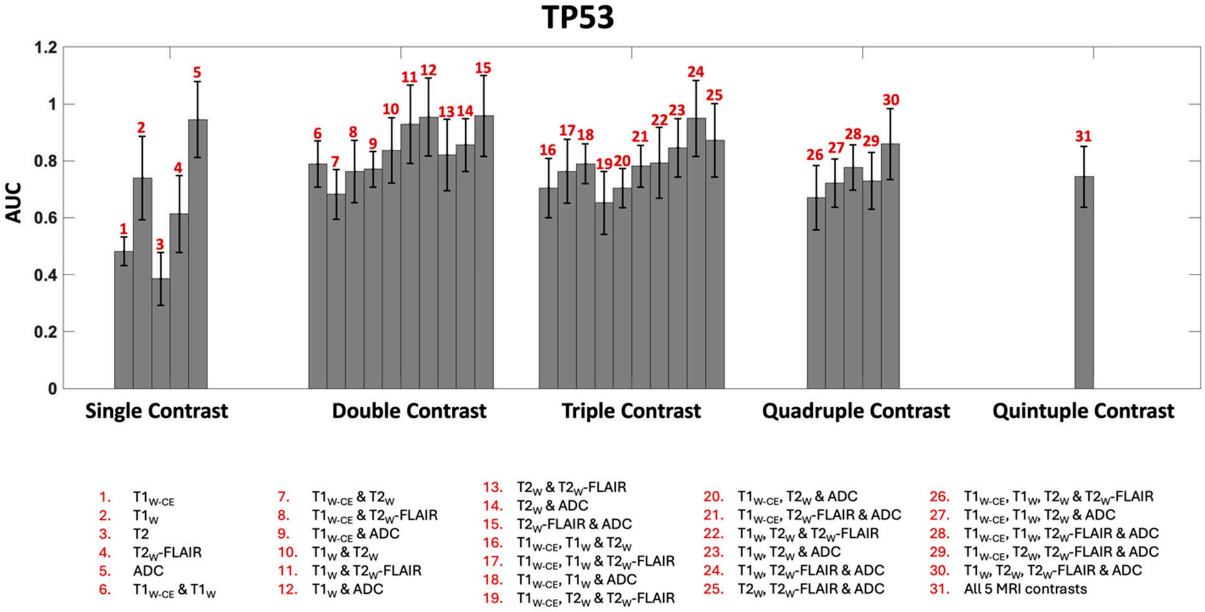

Figure S.3. AUC scores of different MRI contrast combinations for predicting TP53 mutation status with SEM error bars.
(AUC: area under curve; MRI: magnetic resonance imaging; TP53: tumor protein 53; $T1_{W-CE}$: contrast enhanced T1-weighted; $T1_W$: T1-weighted; $T2_W$: T2-weighted; $T2_W$-FLAIR: T2-weighted fluid attenuated inversion recovery; ADC: apparent diffusion coefficient, SEM: standard mean of the error).

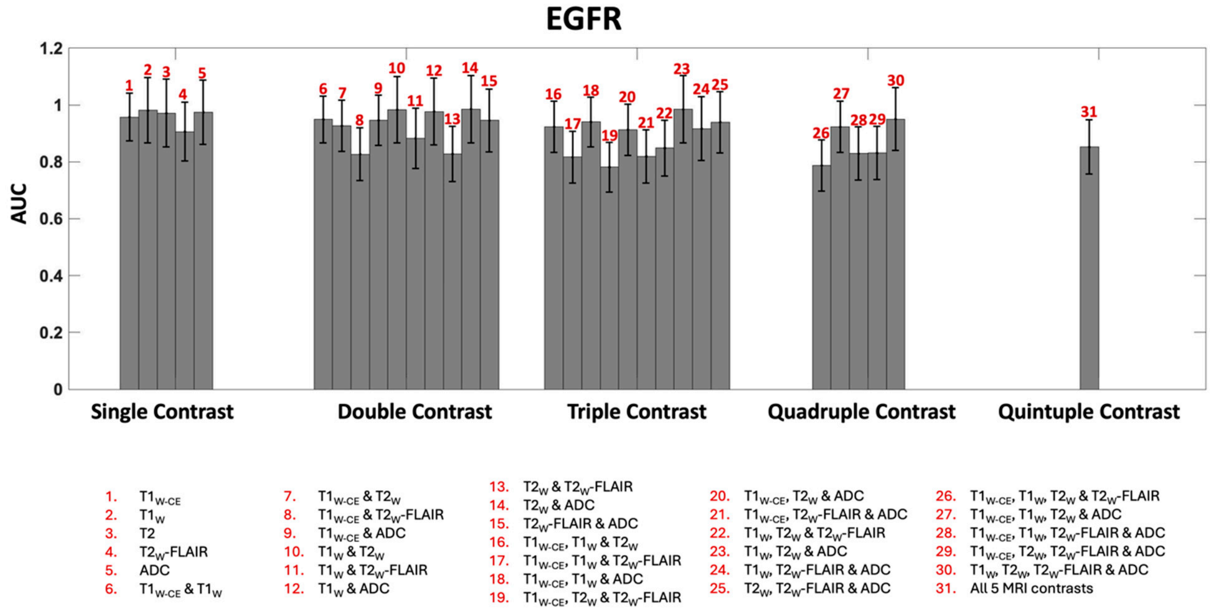

Figure S4. AUC scores of different MRI contrast combinations for predicting EGFR mutation status with SEM error bars.
(AUC: area under curve; MRI: magnetic resonance imaging; EGFR: epidermal growth factor receptor; $T1_{W\text{-}CE}$: contrast enhanced T1-weighted; $T1_W$: T1-weighted; $T2_W$: T2-weighted; $T2_W$-FLAIR: T2-weighted fluid attenuated inversion recovery; ADC: apparent diffusion coefficient, SEM: standard mean of the error).

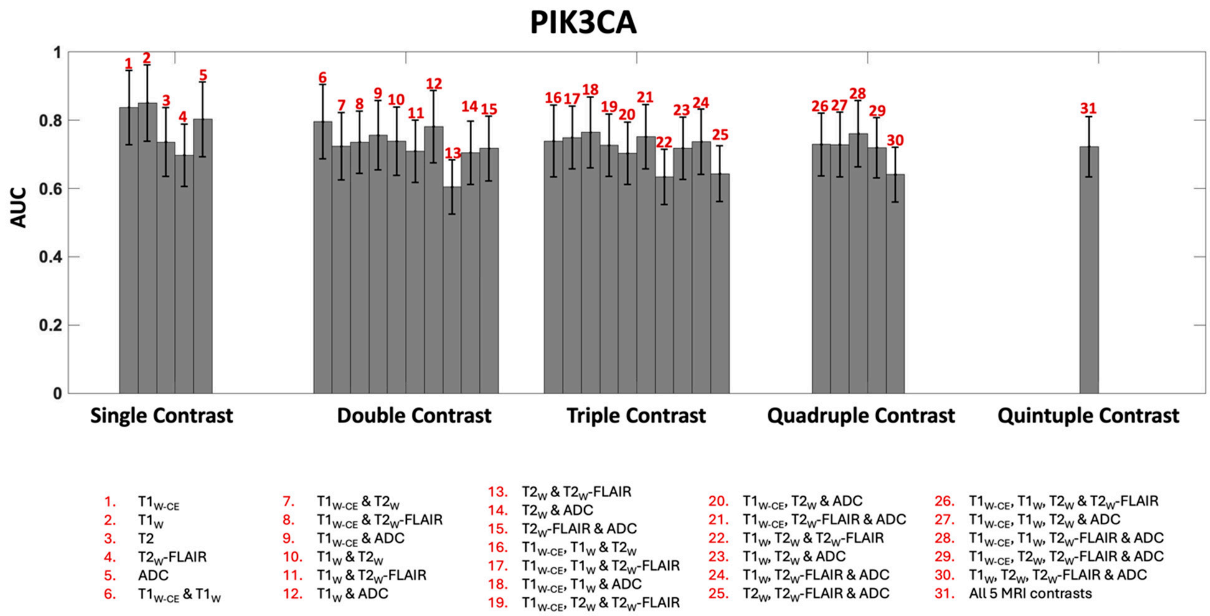

Figure S5. AUC scores of different MRI contrast combinations for predicting PIK3CA mutation status with SEM error bars.

(AUC: area under curve; MRI: magnetic resonance imaging; PIK3CA: phosphatidylinositol-4,5-bisphosphate 3-kinase catalytic subunit alpha; $T1_{W-CE}$: contrast enhanced T1-weighted; $T1_{W}$: T1-weighted; $T2_{W}$: T2-weighted; $T2_{W}$-FLAIR: T2-weighted fluid attenuated inversion recovery; ADC: apparent diffusion coefficient, SEM: standard mean of the error).

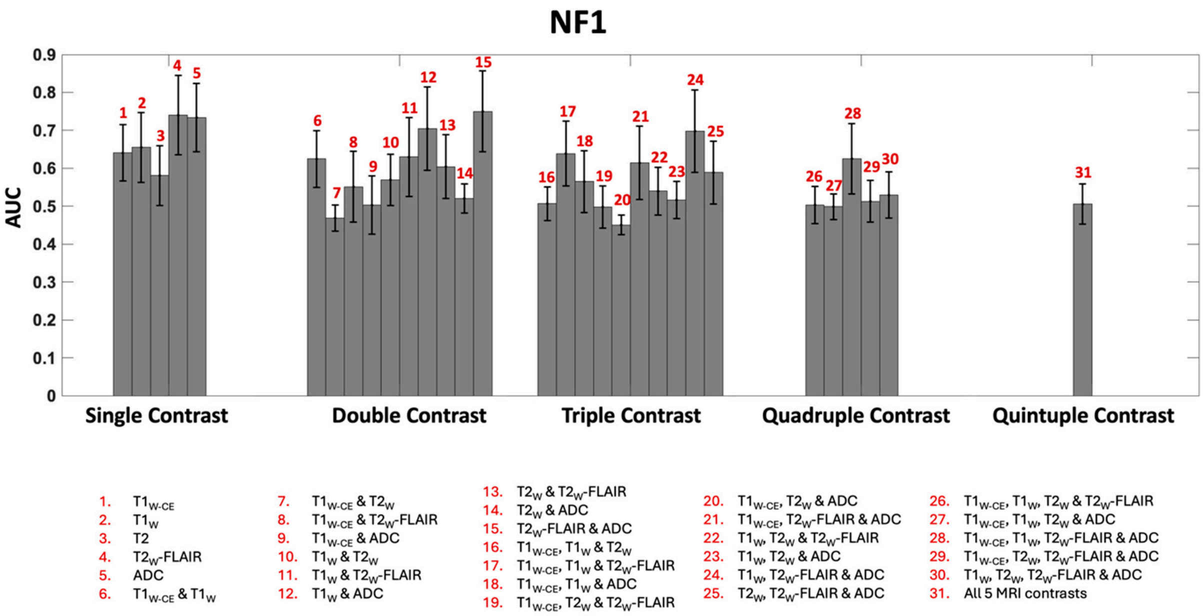

Figure S6. AUC scores of different MRI contrast combinations for predicting NF1 mutation status with SEM error bars.
(AUC: area under curve; MRI: magnetic resonance imaging; NF1: neurofibromatosis type 1; $T1_{W-CE}$: contrast enhanced T1-weighted; $T1_W$: T1-weighted; $T2_W$: T2-weighted; $T2_W$-FLAIR: T2-weighted fluid attenuated inversion recovery; ADC: apparent diffusion coefficient, SEM: standard mean of the error).
